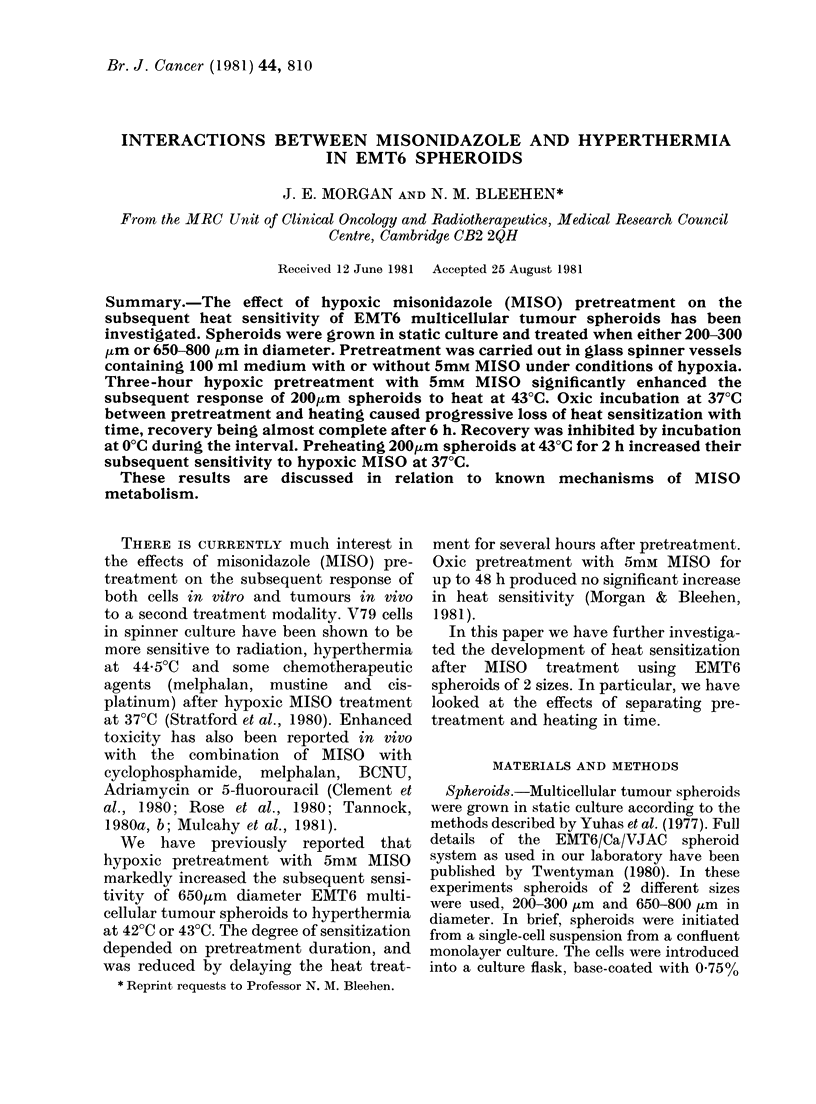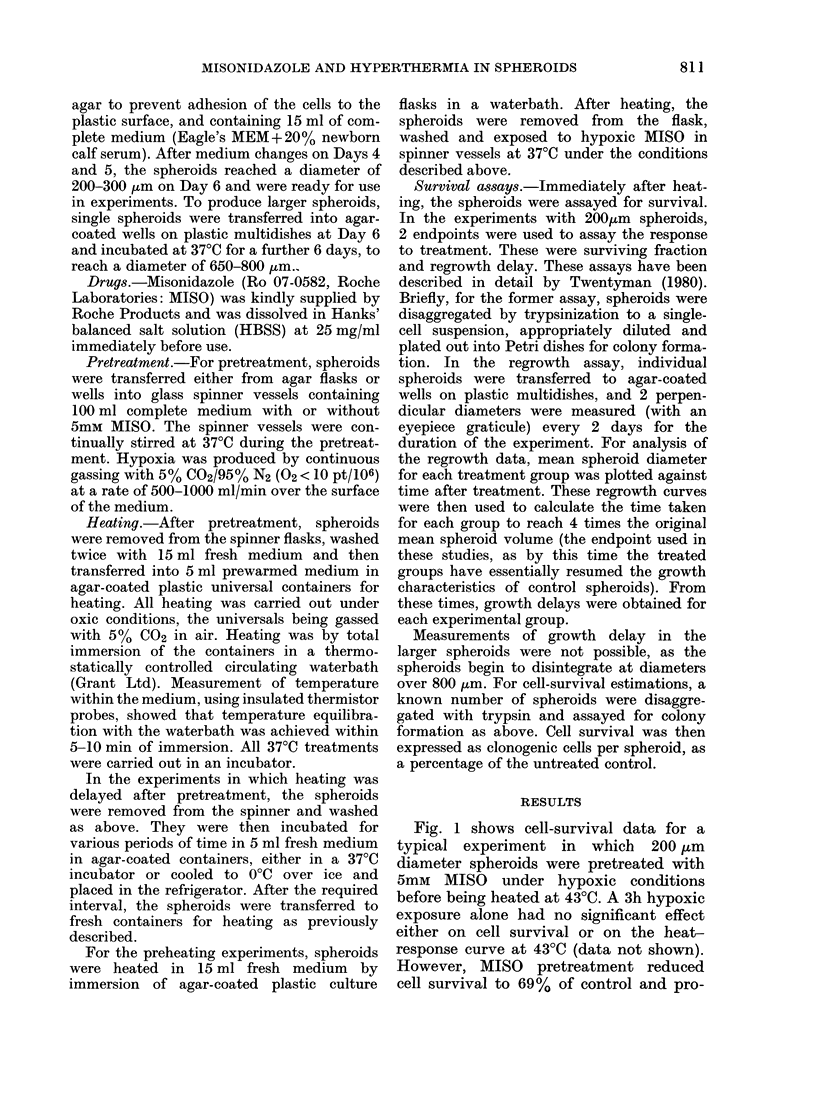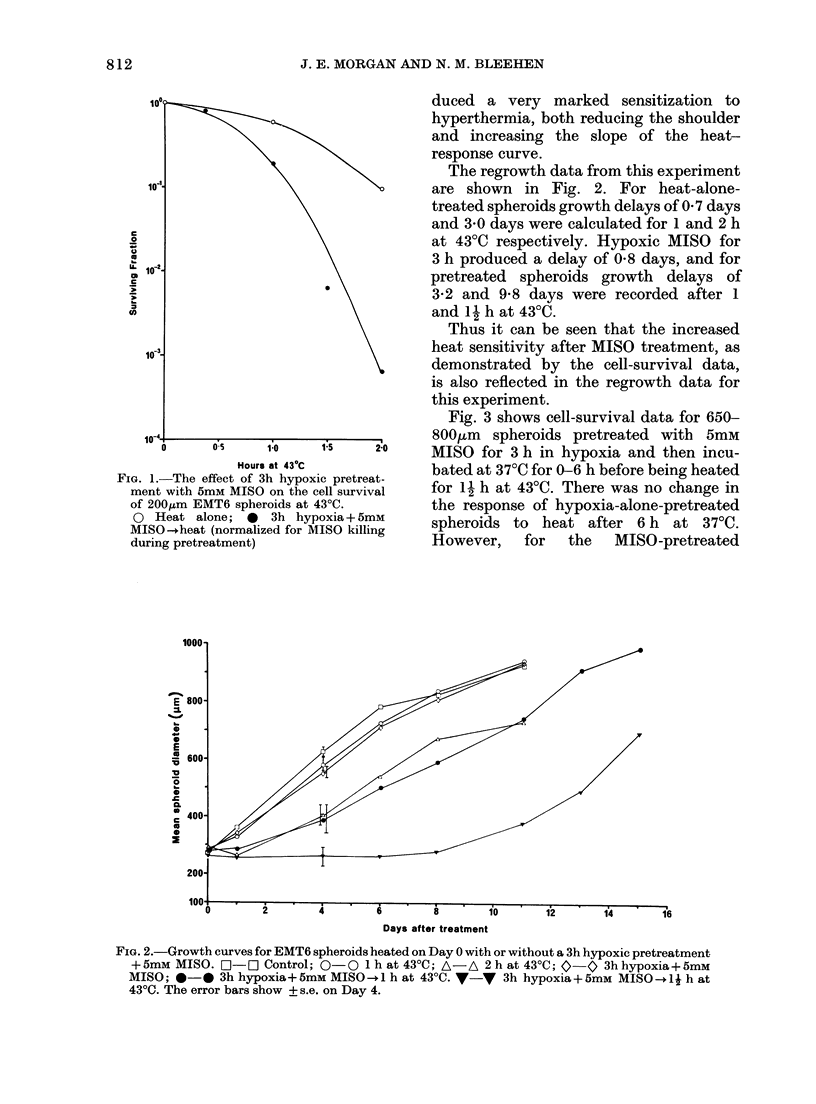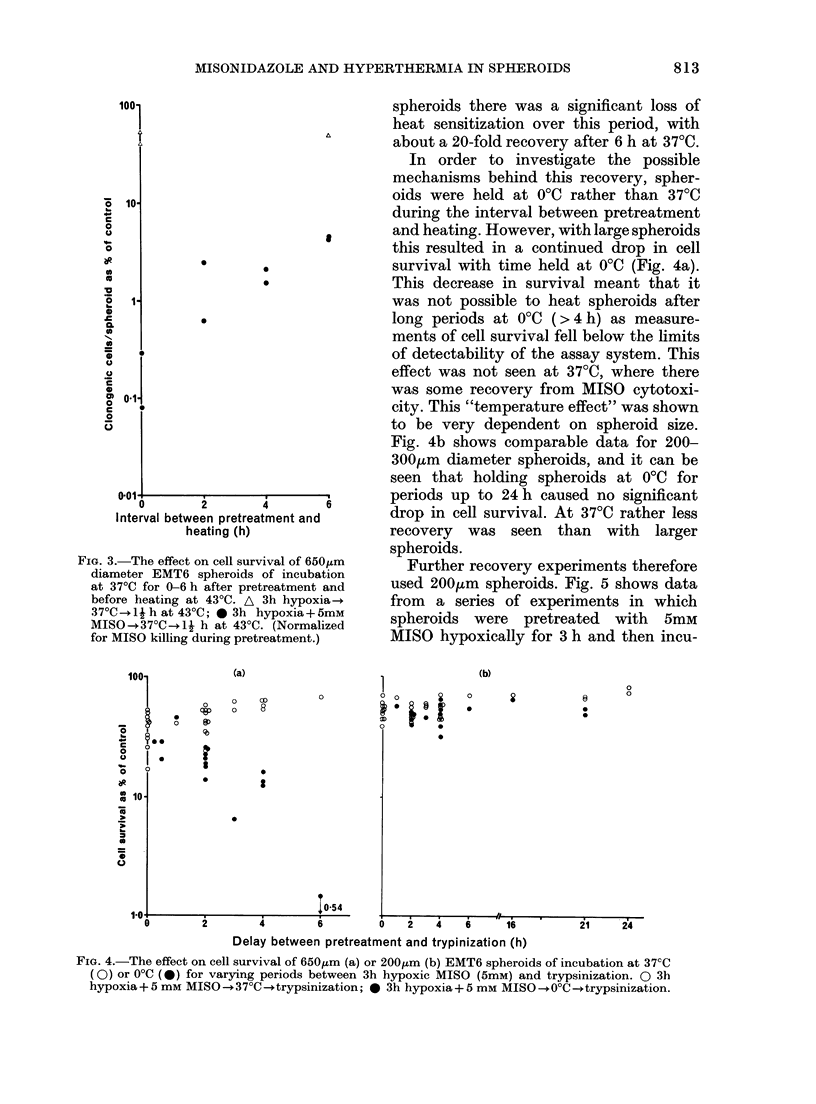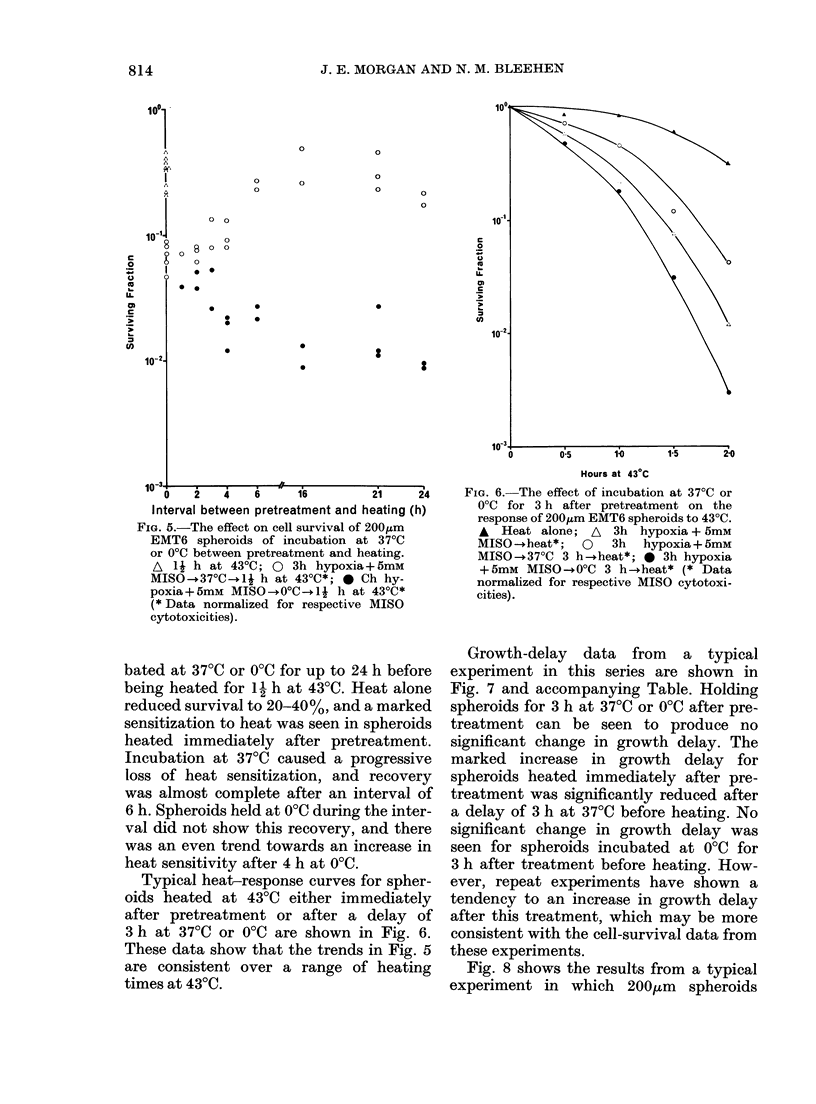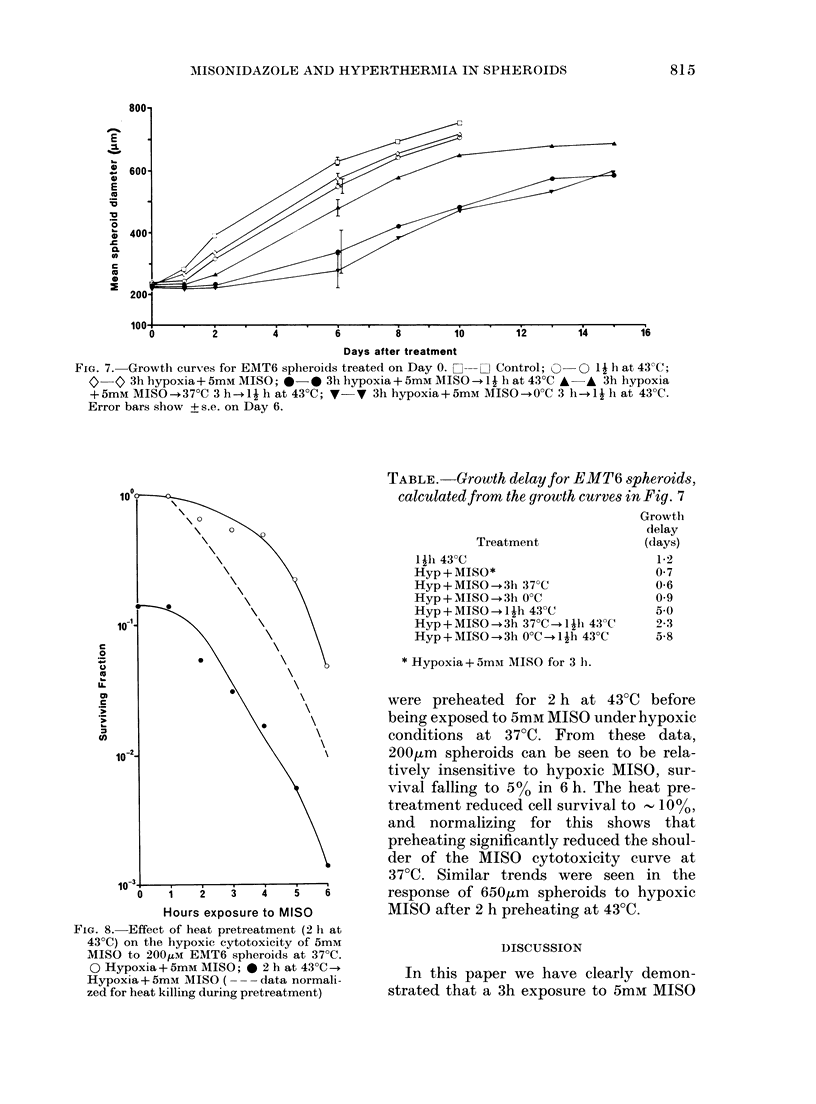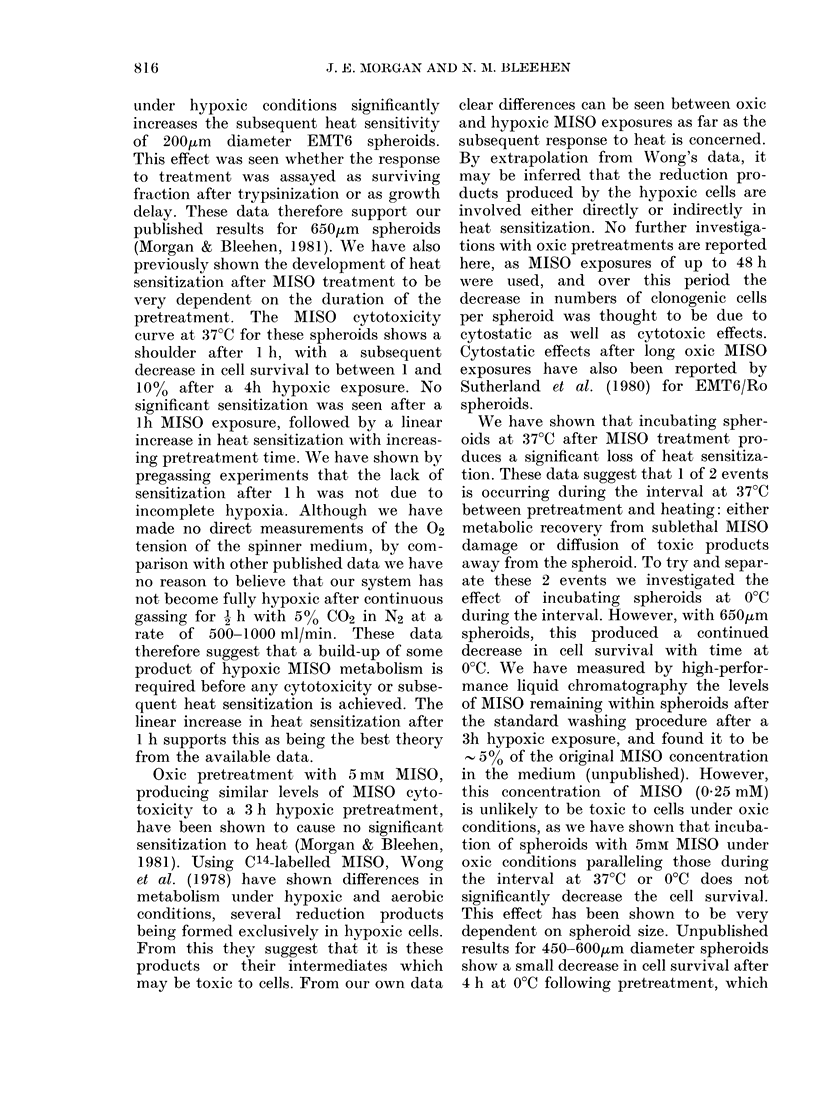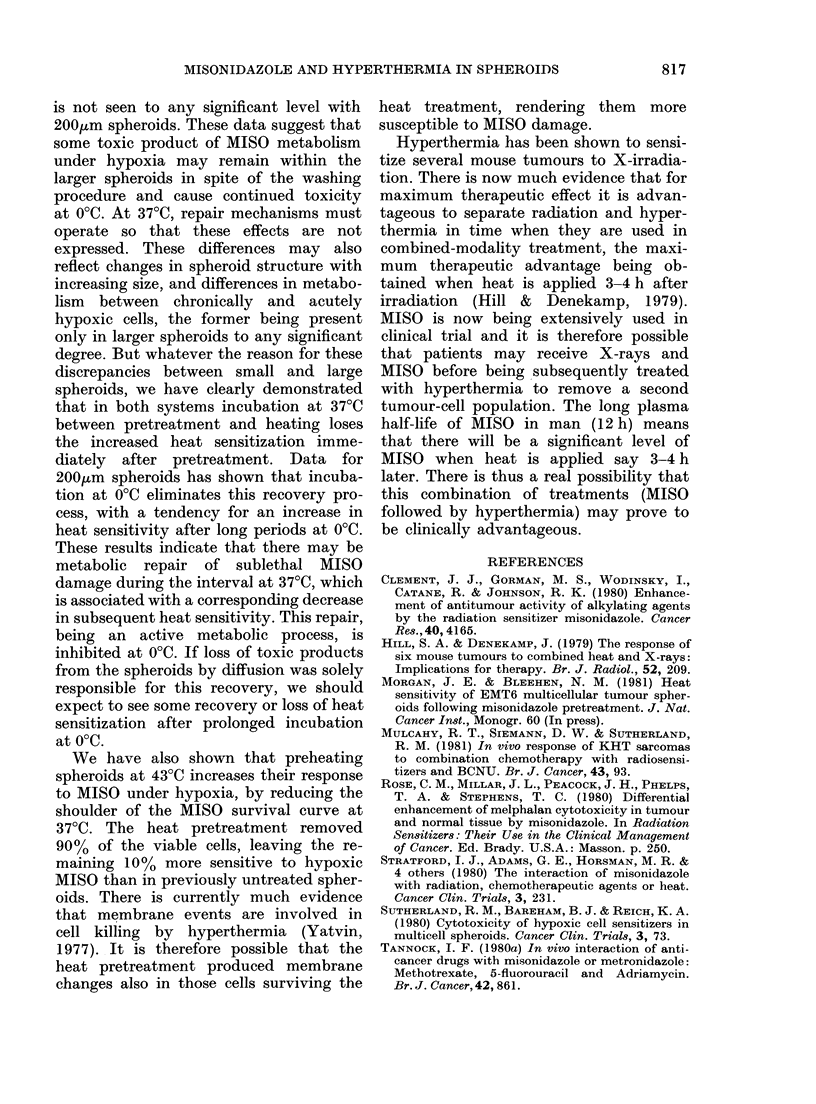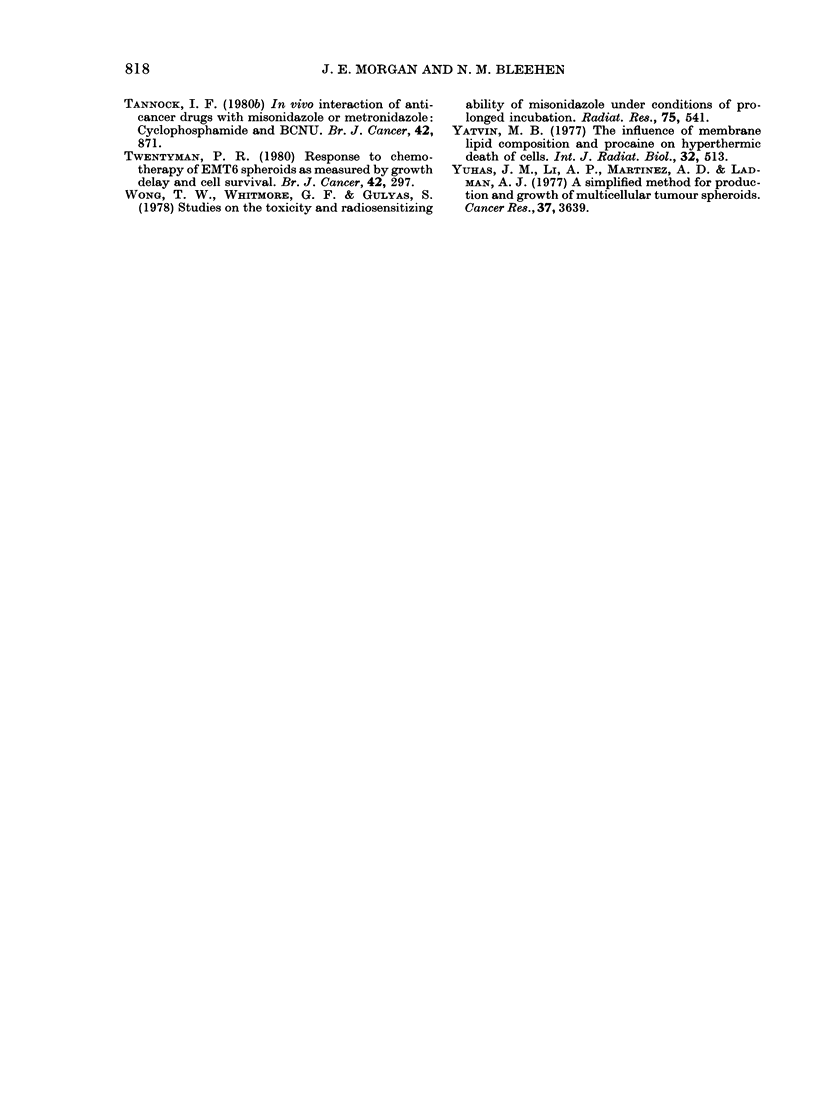# Interactions between misonidazole and hyperthermia in EMT6 spheroids.

**DOI:** 10.1038/bjc.1981.279

**Published:** 1981-12

**Authors:** J. E. Morgan, N. M. Bleehen

## Abstract

The effect of hypoxic misonidazole (MISO) pretreatment on the subsequent heat sensitivity of EMT6 multicellular tumour spheroids has been investigated. Spheroids were grown in static culture and treated when either 200-300 micrometers or 650-800 micrometers in diameter. Pretreatment was carried out in glass spinner vessels containing 100 ml medium with or without 5mM MISO under conditions of hypoxia. Three-hour hypoxic pretreatment with 5mM MISO significantly enhanced the subsequent response of 200 micrometers spheroids to heat at 43 degrees C. Oxic incubation at 37 degrees C between pretreatment and heating caused progressively loss of heat sensitization with time, recovery being almost complete after 6 h. Recovery was inhibited by incubation at 0 degree C during the interval. Preheating 200 micrometers spheroids at 43 degrees C for 2 h increased their subsequent sensitivity to hypoxic MISO at 37 degrees C. These results are discussed in relation to known mechanisms of MISO metabolism.


Br. J. Cancer (1981) 44, 810

### INTERACTIONS BETWEEN MISONIDAZOLE AND HYPERTHERMIA IN EMT6 SPHEROIDS

J. E. MORGAN AND N. M. BLEEHEN*

*From the MRC Unit of Clinical Oncology and Radiotherapeutics, Medical Research Council Centre, Cambridge CB2 2QH*

Received 12 June 1981 Accepted 25 August 1981

**Summary.—The effect of hypoxic misonidazole (MISO) pretreatment on the subsequent heat sensitivity of EMT6 multicellular tumour spheroids has been investigated. Spheroids were grown in static culture and treated when either 200–300 μm or 650–800 μm in diameter. Pretreatment was carried out in glass spinner vessels containing 100 ml medium with or without 5mM MISO under conditions of hypoxia. Three-hour hypoxic pretreatment with 5mM MISO significantly enhanced the subsequent response of 200μm spheroids to heat at 43°C. Oxic incubation at 37°C between pretreatment and heating caused progressive loss of heat sensitization with time, recovery being almost complete after 6 h. Recovery was inhibited by incubation at 0°C during the interval. Preheating 200μm spheroids at 43°C for 2 h increased their subsequent sensitivity to hypoxic MISO at 37°C.**

**These results are discussed in relation to known mechanisms of MISO metabolism.**

THERE IS CURRENTLY much interest in the effects of misonidazole (MISO) pretreatment on the subsequent response of both cells *in vitro* and tumours *in vivo* to a second treatment modality. V79 cells in spinner culture have been shown to be more sensitive to radiation, hyperthermia at 44·5°C and some chemotherapeutic agents (melphalan, mustine and cis-platinum) after hypoxic MISO treatment at 37°C (Stratford *et al.*, 1980). Enhanced toxicity has also been reported *in vivo* with the combination of MISO with cyclophosphamide, melphalan, BCNU, Adriamycin or 5-fluorouracil (Clement *et al.*, 1980; Rose *et al.*, 1980; Tannock, 1980*a*, *b*; Mulcahy *et al.*, 1981).

We have previously reported that hypoxic pretreatment with 5mM MISO markedly increased the subsequent sensitivity of 650μm diameter EMT6 multicellular tumour spheroids to hyperthermia at 42°C or 43°C. The degree of sensitization depended on pretreatment duration, and was reduced by delaying the heat treatment for several hours after pretreatment. Oxic pretreatment with 5mM MISO for up to 48 h produced no significant increase in heat sensitivity (Morgan & Bleehen, 1981).

In this paper we have further investigated the development of heat sensitization after MISO treatment using EMT6 spheroids of 2 sizes. In particular, we have looked at the effects of separating pretreatment and heating in time.

### MATERIALS AND METHODS

*Spheroids.*—Multicellular tumour spheroids were grown in static culture according to the methods described by Yuhas *et al.* (1977). Full details of the EMT6/Ca/VJAC spheroid system as used in our laboratory have been published by Twentyman (1980). In these experiments spheroids of 2 different sizes were used, 200–300 μm and 650–800 μm in diameter. In brief, spheroids were initiated from a single-cell suspension from a confluent monolayer culture. The cells were introduced into a culture flask, base-coated with 0·75%

* Reprint requests to Professor N. M. Bleehen.

agar to prevent adhesion of the cells to the plastic surface, and containing 15 ml of complete medium (Eagle's MEM + 20% newborn calf serum). After medium changes on Days 4 and 5, the spheroids reached a diameter of 200–300 μm on Day 6 and were ready for use in experiments. To produce larger spheroids, single spheroids were transferred into agar-coated wells on plastic multidishes at Day 6 and incubated at 37°C for a further 6 days, to reach a diameter of 650–800 μm.

*Drugs.*—Misonidazole (Ro 07-0582, Roche Laboratories: MISO) was kindly supplied by Roche Products and was dissolved in Hanks' balanced salt solution (HBSS) at 25 mg/ml immediately before use.

*Pretreatment.*—For pretreatment, spheroids were transferred either from agar flasks or wells into glass spinner vessels containing 100 ml complete medium with or without 5mM MISO. The spinner vessels were continually stirred at 37°C during the pretreatment. Hypoxia was produced by continuous gassing with 5% $CO_2$/95% $N_2$ ($O_2 < 10$ pt/$10^6$) at a rate of 500–1000 ml/min over the surface of the medium.

*Heating.*—After pretreatment, spheroids were removed from the spinner flasks, washed twice with 15 ml fresh medium and then transferred into 5 ml prewarmed medium in agar-coated plastic universal containers for heating. All heating was carried out under oxic conditions, the universals being gassed with 5% $CO_2$ in air. Heating was by total immersion of the containers in a thermostatically controlled circulating waterbath (Grant Ltd). Measurement of temperature within the medium, using insulated thermistor probes, showed that temperature equilibration with the waterbath was achieved within 5–10 min of immersion. All 37°C treatments were carried out in an incubator.

In the experiments in which heating was delayed after pretreatment, the spheroids were removed from the spinner and washed as above. They were then incubated for various periods of time in 5 ml fresh medium in agar-coated containers, either in a 37°C incubator or cooled to 0°C over ice and placed in the refrigerator. After the required interval, the spheroids were transferred to fresh containers for heating as previously described.

For the preheating experiments, spheroids were heated in 15 ml fresh medium by immersion of agar-coated plastic culture flasks in a waterbath. After heating, the spheroids were removed from the flask, washed and exposed to hypoxic MISO in spinner vessels at 37°C under the conditions described above.

*Survival assays.*—Immediately after heating, the spheroids were assayed for survival. In the experiments with 200μm spheroids, 2 endpoints were used to assay the response to treatment. These were surviving fraction and regrowth delay. These assays have been described in detail by Twentyman (1980). Briefly, for the former assay, spheroids were disaggregated by trypsinization to a single-cell suspension, appropriately diluted and plated out into Petri dishes for colony formation. In the regrowth assay, individual spheroids were transferred to agar-coated wells on plastic multidishes, and 2 perpendicular diameters were measured (with an eyepiece graticule) every 2 days for the duration of the experiment. For analysis of the regrowth data, mean spheroid diameter for each treatment group was plotted against time after treatment. These regrowth curves were then used to calculate the time taken for each group to reach 4 times the original mean spheroid volume (the endpoint used in these studies, as by this time the treated groups have essentially resumed the growth characteristics of control spheroids). From these times, growth delays were obtained for each experimental group.

Measurements of growth delay in the larger spheroids were not possible, as the spheroids begin to disintegrate at diameters over 800 μm. For cell-survival estimations, a known number of spheroids were disaggregated with trypsin and assayed for colony formation as above. Cell survival was then expressed as clonogenic cells per spheroid, as a percentage of the untreated control.

### RESULTS

Fig. 1 shows cell-survival data for a typical experiment in which 200 μm diameter spheroids were pretreated with 5mM MISO under hypoxic conditions before being heated at 43°C. A 3h hypoxic exposure alone had no significant effect either on cell survival or on the heat–response curve at 43°C (data not shown). However, MISO pretreatment reduced cell survival to 69% of control and pro-

FIG. 1.—The effect of 3h hypoxic pretreatment with 5mM MISO on the cell survival of 200$\mu$m EMT6 spheroids at 43°C.
○ Heat alone; ● 3h hypoxia + 5mM MISO→heat (normalized for MISO killing during pretreatment)

duced a very marked sensitization to hyperthermia, both reducing the shoulder and increasing the slope of the heat–response curve.

The regrowth data from this experiment are shown in Fig. 2. For heat-alone-treated spheroids growth delays of 0·7 days and 3·0 days were calculated for 1 and 2 h at 43°C respectively. Hypoxic MISO for 3 h produced a delay of 0·8 days, and for pretreated spheroids growth delays of 3·2 and 9·8 days were recorded after 1 and $1\frac{1}{2}$ h at 43°C.

Thus it can be seen that the increased heat sensitivity after MISO treatment, as demonstrated by the cell-survival data, is also reflected in the regrowth data for this experiment.

Fig. 3 shows cell-survival data for 650–800$\mu$m spheroids pretreated with 5mM MISO for 3 h in hypoxia and then incubated at 37°C for 0–6 h before being heated for $1\frac{1}{2}$ h at 43°C. There was no change in the response of hypoxia-alone-pretreated spheroids to heat after 6 h at 37°C. However, for the MISO-pretreated

FIG. 2.—Growth curves for EMT6 spheroids heated on Day 0 with or without a 3h hypoxic pretreatment + 5mM MISO. □—□ Control; ○—○ 1 h at 43°C; △—△ 2 h at 43°C; ◇—◇ 3h hypoxia + 5mM MISO; ●—● 3h hypoxia + 5mM MISO→1 h at 43°C. ▼—▼ 3h hypoxia + 5mM MISO→$1\frac{1}{2}$ h at 43°C. The error bars show ±s.e. on Day 4.

FIG. 3.—The effect on cell survival of 650$\mu$m diameter EMT6 spheroids of incubation at 37°C for 0–6 h after pretreatment and before heating at 43°C. △ 3h hypoxia→ 37°C→$1\frac{1}{2}$ h at 43°C; ● 3h hypoxia+5mM MISO→37°C→$1\frac{1}{2}$ h at 43°C. (Normalized for MISO killing during pretreatment.)

spheroids there was a significant loss of heat sensitization over this period, with about a 20-fold recovery after 6 h at 37°C.

In order to investigate the possible mechanisms behind this recovery, spheroids were held at 0°C rather than 37°C during the interval between pretreatment and heating. However, with large spheroids this resulted in a continued drop in cell survival with time held at 0°C (Fig. 4a). This decrease in survival meant that it was not possible to heat spheroids after long periods at 0°C (>4 h) as measurements of cell survival fell below the limits of detectability of the assay system. This effect was not seen at 37°C, where there was some recovery from MISO cytotoxicity. This "temperature effect" was shown to be very dependent on spheroid size. Fig. 4b shows comparable data for 200–300$\mu$m diameter spheroids, and it can be seen that holding spheroids at 0°C for periods up to 24 h caused no significant drop in cell survival. At 37°C rather less recovery was seen than with larger spheroids.

Further recovery experiments therefore used 200$\mu$m spheroids. Fig. 5 shows data from a series of experiments in which spheroids were pretreated with 5mM MISO hypoxically for 3 h and then incu-

FIG. 4.—The effect on cell survival of 650$\mu$m (a) or 200$\mu$m (b) EMT6 spheroids of incubation at 37°C (○) or 0°C (●) for varying periods between 3h hypoxic MISO (5mM) and trypsinization. ○ 3h hypoxia+5 mM MISO→37°C→trypsinization; ● 3h hypoxia+5 mM MISO→0°C→trypsinization.

FIG. 5.—The effect on cell survival of 200$\mu$m EMT6 spheroids of incubation at 37°C or 0°C between pretreatment and heating. △ $1\frac{1}{2}$ h at 43°C; ○ 3h hypoxia + 5mM MISO→37°C→$1\frac{1}{2}$ h at 43°C*; ● Ch hypoxia + 5mM MISO→0°C→$1\frac{1}{2}$ h at 43°C* (* Data normalized for respective MISO cytotoxicities).

FIG. 6.—The effect of incubation at 37°C or 0°C for 3 h after pretreatment on the response of 200$\mu$m EMT6 spheroids to 43°C. ▲ Heat alone; △ 3h hypoxia + 5mM MISO→heat*; ○ 3h hypoxia + 5mM MISO→37°C 3 h→heat*; ● 3h hypoxia + 5mM MISO→0°C 3 h→heat* (* Data normalized for respective MISO cytotoxicities).

bated at 37°C or 0°C for up to 24 h before being heated for $1\frac{1}{2}$ h at 43°C. Heat alone reduced survival to 20–40%, and a marked sensitization to heat was seen in spheroids heated immediately after pretreatment. Incubation at 37°C caused a progressive loss of heat sensitization, and recovery was almost complete after an interval of 6 h. Spheroids held at 0°C during the interval did not show this recovery, and there was an even trend towards an increase in heat sensitivity after 4 h at 0°C.

Typical heat–response curves for spheroids heated at 43°C either immediately after pretreatment or after a delay of 3 h at 37°C or 0°C are shown in Fig. 6. These data show that the trends in Fig. 5 are consistent over a range of heating times at 43°C.

Growth-delay data from a typical experiment in this series are shown in Fig. 7 and accompanying Table. Holding spheroids for 3 h at 37°C or 0°C after pretreatment can be seen to produce no significant change in growth delay. The marked increase in growth delay for spheroids heated immediately after pretreatment was significantly reduced after a delay of 3 h at 37°C before heating. No significant change in growth delay was seen for spheroids incubated at 0°C for 3 h after treatment before heating. However, repeat experiments have shown a tendency to an increase in growth delay after this treatment, which may be more consistent with the cell-survival data from these experiments.

Fig. 8 shows the results from a typical experiment in which 200$\mu$m spheroids

FIG. 7.—Growth curves for EMT6 spheroids treated on Day 0. □—□ Control; ○—○ 1½ h at 43°C; ◇—◇ 3h hypoxia + 5mM MISO; ●—● 3h hypoxia + 5mM MISO→1½ h at 43°C ▲—▲ 3h hypoxia + 5mM MISO→37°C 3 h→1½ h at 43°C; ▼—▼ 3h hypoxia + 5mM MISO→0°C 3 h→1½ h at 43°C. Error bars show ±s.e. on Day 6.

FIG. 8.—Effect of heat pretreatment (2 h at 43°C) on the hypoxic cytotoxicity of 5mM MISO to 200μM EMT6 spheroids at 37°C. ○ Hypoxia + 5mM MISO; ● 2 h at 43°C→ Hypoxia + 5mM MISO (– – – data normalized for heat killing during pretreatment)

TABLE.—*Growth delay for EMT6 spheroids, calculated from the growth curves in Fig. 7*

| Treatment | Growth delay (days) |
|---|---|
| 1½h 43°C | 1·2 |
| Hyp + MISO* | 0·7 |
| Hyp + MISO→3h 37°C | 0·6 |
| Hyp + MISO→3h 0°C | 0·9 |
| Hyp + MISO→1½h 43°C | 5·0 |
| Hyp + MISO→3h 37°C→1½h 43°C | 2·3 |
| Hyp + MISO→3h 0°C→1½h 43°C | 5·8 |

* Hypoxia + 5mM MISO for 3 h.

were preheated for 2 h at 43°C before being exposed to 5mM MISO under hypoxic conditions at 37°C. From these data, 200μm spheroids can be seen to be relatively insensitive to hypoxic MISO, survival falling to 5% in 6 h. The heat pretreatment reduced cell survival to ~10%, and normalizing for this shows that preheating significantly reduced the shoulder of the MISO cytotoxicity curve at 37°C. Similar trends were seen in the response of 650μm spheroids to hypoxic MISO after 2 h preheating at 43°C.

### DISCUSSION

In this paper we have clearly demonstrated that a 3h exposure to 5mM MISO

under hypoxic conditions significantly increases the subsequent heat sensitivity of 200$\mu$m diameter EMT6 spheroids. This effect was seen whether the response to treatment was assayed as surviving fraction after trypsinization or as growth delay. These data therefore support our published results for 650$\mu$m spheroids (Morgan & Bleehen, 1981). We have also previously shown the development of heat sensitization after MISO treatment to be very dependent on the duration of the pretreatment. The MISO cytotoxicity curve at 37°C for these spheroids shows a shoulder after 1 h, with a subsequent decrease in cell survival to between 1 and 10% after a 4h hypoxic exposure. No significant sensitization was seen after a 1h MISO exposure, followed by a linear increase in heat sensitization with increasing pretreatment time. We have shown by pregassing experiments that the lack of sensitization after 1 h was not due to incomplete hypoxia. Although we have made no direct measurements of the $O_2$ tension of the spinner medium, by comparison with other published data we have no reason to believe that our system has not become fully hypoxic after continuous gassing for $\frac{1}{2}$ h with 5% $CO_2$ in $N_2$ at a rate of 500–1000 ml/min. These data therefore suggest that a build-up of some product of hypoxic MISO metabolism is required before any cytotoxicity or subsequent heat sensitization is achieved. The linear increase in heat sensitization after 1 h supports this as being the best theory from the available data.

Oxic pretreatment with 5 mM MISO, producing similar levels of MISO cytotoxicity to a 3 h hypoxic pretreatment, have been shown to cause no significant sensitization to heat (Morgan & Bleehen, 1981). Using $C^{14}$-labelled MISO, Wong *et al.* (1978) have shown differences in metabolism under hypoxic and aerobic conditions, several reduction products being formed exclusively in hypoxic cells. From this they suggest that it is these products or their intermediates which may be toxic to cells. From our own data clear differences can be seen between oxic and hypoxic MISO exposures as far as the subsequent response to heat is concerned. By extrapolation from Wong's data, it may be inferred that the reduction products produced by the hypoxic cells are involved either directly or indirectly in heat sensitization. No further investigations with oxic pretreatments are reported here, as MISO exposures of up to 48 h were used, and over this period the decrease in numbers of clonogenic cells per spheroid was thought to be due to cytostatic as well as cytotoxic effects. Cytostatic effects after long oxic MISO exposures have also been reported by Sutherland *et al.* (1980) for EMT6/Ro spheroids.

We have shown that incubating spheroids at 37°C after MISO treatment produces a significant loss of heat sensitization. These data suggest that 1 of 2 events is occurring during the interval at 37°C between pretreatment and heating: either metabolic recovery from sublethal MISO damage or diffusion of toxic products away from the spheroid. To try and separate these 2 events we investigated the effect of incubating spheroids at 0°C during the interval. However, with 650$\mu$m spheroids, this produced a continued decrease in cell survival with time at 0°C. We have measured by high-performance liquid chromatography the levels of MISO remaining within spheroids after the standard washing procedure after a 3h hypoxic exposure, and found it to be ~5% of the original MISO concentration in the medium (unpublished). However, this concentration of MISO (0·25 mM) is unlikely to be toxic to cells under oxic conditions, as we have shown that incubation of spheroids with 5mM MISO under oxic conditions paralleling those during the interval at 37°C or 0°C does not significantly decrease the cell survival. This effect has been shown to be very dependent on spheroid size. Unpublished results for 450–600$\mu$m diameter spheroids show a small decrease in cell survival after 4 h at 0°C following pretreatment, which is not seen to any significant level with 200$\mu$m spheroids. These data suggest that some toxic product of MISO metabolism under hypoxia may remain within the larger spheroids in spite of the washing procedure and cause continued toxicity at 0°C. At 37°C, repair mechanisms must operate so that these effects are not expressed. These differences may also reflect changes in spheroid structure with increasing size, and differences in metabolism between chronically and acutely hypoxic cells, the former being present only in larger spheroids to any significant degree. But whatever the reason for these discrepancies between small and large spheroids, we have clearly demonstrated that in both systems incubation at 37°C between pretreatment and heating loses the increased heat sensitization immediately after pretreatment. Data for 200$\mu$m spheroids has shown that incubation at 0°C eliminates this recovery process, with a tendency for an increase in heat sensitivity after long periods at 0°C. These results indicate that there may be metabolic repair of sublethal MISO damage during the interval at 37°C, which is associated with a corresponding decrease in subsequent heat sensitivity. This repair, being an active metabolic process, is inhibited at 0°C. If loss of toxic products from the spheroids by diffusion was solely responsible for this recovery, we should expect to see some recovery or loss of heat sensitization after prolonged incubation at 0°C.

We have also shown that preheating spheroids at 43°C increases their response to MISO under hypoxia, by reducing the shoulder of the MISO survival curve at 37°C. The heat pretreatment removed 90% of the viable cells, leaving the remaining 10% more sensitive to hypoxic MISO than in previously untreated spheroids. There is currently much evidence that membrane events are involved in cell killing by hyperthermia (Yatvin, 1977). It is therefore possible that the heat pretreatment produced membrane changes also in those cells surviving the heat treatment, rendering them more susceptible to MISO damage.

Hyperthermia has been shown to sensitize several mouse tumours to X-irradiation. There is now much evidence that for maximum therapeutic effect it is advantageous to separate radiation and hyperthermia in time when they are used in combined-modality treatment, the maximum therapeutic advantage being obtained when heat is applied 3–4 h after irradiation (Hill & Denekamp, 1979). MISO is now being extensively used in clinical trial and it is therefore possible that patients may receive X-rays and MISO before being subsequently treated with hyperthermia to remove a second tumour-cell population. The long plasma half-life of MISO in man (12 h) means that there will be a significant level of MISO when heat is applied say 3–4 h later. There is thus a real possibility that this combination of treatments (MISO followed by hyperthermia) may prove to be clinically advantageous.

### REFERENCES

Clement, J. J., Gorman, M. S., Wodinsky, I., Catane, R. & Johnson, R. K. (1980) Enhancement of antitumour activity of alkylating agents by the radiation sensitizer misonidazole. *Cancer Res.*, **40**, 4165.

Hill, S. A. & Denekamp, J. (1979) The response of six mouse tumours to combined heat and X-rays: Implications for therapy. *Br. J. Radiol.*, **52**, 209.

Morgan, J. E. & Bleehen, N. M. (1981) Heat sensitivity of EMT6 multicellular tumour spheroids following misonidazole pretreatment. *J. Nat. Cancer Inst.*, Monogr. 60 (In press).

Mulcahy, R. T., Siemann, D. W. & Sutherland, R. M. (1981) *In vivo* response of KHT sarcomas to combination chemotherapy with radiosensitizers and BCNU. *Br. J. Cancer*, **43**, 93.

Rose, C. M., Millar, J. L., Peacock, J. H., Phelps, T. A. & Stephens, T. C. (1980) Differential enhancement of melphalan cytotoxicity in tumour and normal tissue by misonidazole. In *Radiation Sensitizers: Their Use in the Clinical Management of Cancer*. Ed. Brady. U.S.A.: Masson. p. 250.

Stratford, I. J., Adams, G. E., Horsman, M. R. & 4 others (1980) The interaction of misonidazole with radiation, chemotherapeutic agents or heat. *Cancer Clin. Trials*, **3**, 231.

Sutherland, R. M., Bareham, B. J. & Reich, K. A. (1980) Cytotoxicity of hypoxic cell sensitizers in multicell spheroids. *Cancer Clin. Trials*, **3**, 73.

Tannock, I. F. (1980*a*) *In vivo* interaction of anticancer drugs with misonidazole or metronidazole: Methotrexate, 5-fluorouracil and Adriamycin. *Br. J. Cancer*, **42**, 861.

TANNOCK, I. F. (1980*b*) *In vivo* interaction of anticancer drugs with misonidazole or metronidazole: Cyclophosphamide and BCNU. *Br. J. Cancer*, **42**, 871.

TWENTYMAN, P. R. (1980) Response to chemotherapy of EMT6 spheroids as measured by growth delay and cell survival. *Br. J. Cancer*, **42**, 297.

WONG, T. W., WHITMORE, G. F. & GULYAS, S. (1978) Studies on the toxicity and radiosensitizing ability of misonidazole under conditions of prolonged incubation. *Radiat. Res.*, **75**, 541.

YATVIN, M. B. (1977) The influence of membrane lipid composition and procaine on hyperthermic death of cells. *Int. J. Radiat. Biol.*, **32**, 513.

YUHAS, J. M., LI, A. P., MARTINEZ, A. D. & LADMAN, A. J. (1977) A simplified method for production and growth of multicellular tumour spheroids. *Cancer Res.*, **37**, 3639.